# Increasing salinity drastically reduces hatching success of crustaceans from depression wetlands of the semi-arid Eastern Cape Karoo region, South Africa

**DOI:** 10.1038/s41598-018-24137-0

**Published:** 2018-04-13

**Authors:** Annah Mabidi, Matthew S. Bird, Renzo Perissinotto

**Affiliations:** 1 South African Research Chairs Initiative, Shallow Water Ecosystems, Nelson Mandela University, P.O. Box 77000, Port Elizabeth, 6031 South Africa; 2 Africa Earth Observatory Network, Nelson Mandela University, P.O. Box 77000, Port Elizabeth, 6031 South Africa; 3 Department of Zoology, University of Johannesburg, P.O. Box, 524, Auckland Park, Johannesburg, 2006 South Africa

## Abstract

Salinity is an important factor affecting freshwater aquatic species distribution and diversity. The semi-arid Eastern Cape Karoo region of South Africa has been earmarked for shale gas development through hydraulic fracturing. The process uses large amounts of water and produces briny wastewater. When not managed properly, these wastewaters may lead to salinisation of surface freshwater bodies in the region. Therefore, the effect of salinity on the hatching success of crustacean resting eggs was examined using sediments from four depression wetlands found in the region. The sediments were exposed for 28 days to salinity levels of 0.5 g L^−1^, 2.5 g L^−1^, 5 g L^−1^ and 10 g L^−1^. Control aquaria in which no salt was added were also set up. There was a significant decrease in the emerged taxa richness and abundances at salinities of 2.5 g L^−1^ and above. Anostraca, Notostraca and Spinicaudata hatchlings were abundant at salinities of 0.5 g L^−1^ and below, while Copepoda, Daphniidae (Cladocera) and Ostracoda were observed in the highest salinity, but their densities were still lower with increased salinities. Given the importance of large branchiopods in the trophic balance of depression wetlands, their loss may alter the ecological balance and function of these ecosystems.

## Introduction

Salinisation is recognized globally as a major threat to freshwater resources^1–4^. Dissolved salts occur naturally in the aquatic environment, however at high concentrations salts are toxicants^5,6^. Salt enters aquatic systems from groundwater, from terrestrial material via the weathering of rocks, from the atmosphere, through transportation by wind and rain or through anthropogenic inputs and climatic processes^2,7^. Increased salinisation resulting from land-use change is most likely to occur in semi-arid or arid regions of the world^8,9^. Wetlands in these areas occasionally experience periods of higher salinity under natural conditions, because of the high evaporative conditions and the variability of inflows^9^. However, these wetlands are now often experiencing extended periods of high salinity due to the impacts associated with changes in land cover and land use^10^.

The Eastern Cape Karoo region of South Africa has recently been earmarked for shale gas exploration using the hydraulic fracturing technique^11,12^. This process involves high amounts of water and disposal of briny wastewater^13^, which when not managed properly may find its way into freshwater systems. This region is already experiencing increasing aridity due to climate change^14,15^, thus shale gas development through hydraulic fracturing may compound the problems of salinisation of freshwater systems in the region. Freshwater is generally defined as water in which salinity is less than 3 g L^−1 ^^16^ and most freshwater biota do not tolerate large increases in salt concentrations^17^. Increasing salinity may cause direct physiological changes, resulting in loss of some species, while indirect changes occur where increasing salinity modifies community structure and function by removing or adding taxa that provide refuge, food or modify predation pressure^3^. Brock, *et al*.^16^ stated that there is potential for increasing salinity in freshwater rivers and wetlands to decrease the species richness of aquatic communities, resulting in loss of wetland biodiversity. At most risk of the effects of salinisation are biota of temporary depression wetlands. Depression wetlands are shallow basins, often endorheic, that allow the accumulation of surface water thereby remaining submerged for sufficiently long periods to allow the development of animal communities^18,19^. Their relatively small volume and shallow depth mean that they are easily degraded by pollution and land drainage^20,21^. Endorheic wetlands are potentially at higher risk of salinisation than flow-through systems, as there is no salt removal mechanism within them^9^.

Branchiopod crustaceans, as permanent inhabitants of temporary depression wetlands, have adapted to the often-harsh conditions of this environment through reproductive characteristics that include early hatching, rapid maturation and early start of egg production during the wet phase, as well as production of dormant life stages during the dry phase^22–24^. The highly resistant life stages, such as resting eggs or diapausing life stages, are capable of surviving long periods of drought^25,26^. These dormant eggs accumulate in the sediment resulting in the formation of an egg bank^25^. The eggs remain dormant until cues such as temperature, light and conductivity induce hatching. Emergence of hatchlings from the sediments can be extended over several days, even under favourable conditions. This is a bet hedging strategy employed by many inhabitants of temporary waters and ensures that not all viable resting eggs emerge before the hydroperiod has become properly established^27^. Previous research has shown that conductivity is one of the most important cues for the hatching of dormant eggs of branchiopod crustaceans^26,28^ and increases in salinity may inhibit emergence from resting eggs^16,29^. Higher salinities are known to be a limiting factor particularly for hatching of resting eggs^26^, disrupt early life stages of eggs and juveniles^30,31^ and affect the long-term viability of dormant invertebrate eggs^17^.

Salinisation of freshwater systems is thus often detrimental to freshwater ecosystems^32^ and can lead to major changes in the community composition of freshwater wetlands^16,33^. For example, Nielsen, *et al*.^34^ found that salinities between 1000 and 5000 mg L^−1^ decreased the species richness and abundance of organisms found in an intermittent temporary wetland on the New England Tablelands and a semi-permanent wetland on the River Murray floodplain in Australia. The aim of our study is to investigate how hatchling abundances and richness of crustacean resting eggs are affected by increasing salinity for a series of depression wetlands in the Eastern Cape Karoo region, using a controlled salinity exposure experiment. We hypothesised that increasing salinity to above 1 g L^−1^ will reduce the hatching success of resting eggs, as found by Hart, *et al*.^30^. This information can be used to estimate the region’s temporary wetland ecosystem sensitivity in the face of the imminent onset of hydraulic fracturing operations in the region.

## Results

### Environmental variables

The geographical location of the depression wetlands and the key variables measured during the 2014/2015 wet season sampling (November and April representing periods of maximum precipitation) are presented in Supplementary Table S1. The mean conductivity values measured in the control aquaria were 0.08, 0.05 and 0.05 mS cm^−1^ for W25, W27 and W27B, respectively (Table 1). These were within the ranges measured in the four depression wetlands during the 2014/2015 wet season sampling events (Supplementary Table S1), except for W110 which had a lower mean value than those recorded during the two wet season sampling events (Table 1). The measured pH values ranged between 7 and 9 across all treatments for the four depressions (Table 1) and these values were within the ranges measured during the 2014/2015 wet season sampling event (Supplementary Table S1). More details of the biogeochemical characteristics (including conductivity and pH) of the study area are given in Mabidi, *et al*.^35^.

**Table 1:** Environmental variables (mean and standard deviation) measured in the aquaria at five salinity treatments.

Wetland code	Variable	Control	0.5 g L^−1^	2.5 g L^−1^	5 g L^−1^	10 g L^−1^
W25	Temperature (°C)	19.55 ± 0.21	19.49 ± 0.23	19.54 ± 0.18	19.55 ± 0.15	19.60 ± 0.14
	pH	8.64 ± 0.78	8.24 ± 0.70	8.06 ± 0.67	8.04 ± 0.67	8.19 ± 0.70
	Conductivity (mS cm^−1^)	0.08 ± 0.06	1.30 ± 0.20	4.57 ± 0.71	9.17 ± 1.41	16.34 ± 1.36
W27	Temperature (°C)	19.53 ± 0.18	19.50 ± 0.16	19.50 ± 0.15	19.57 ± 0.11	19.58 ± 0.19
	pH	8.28 ± 0.84	7.97 ± 0.63	7.77 ± 0.60	7.65 ± 0.61	7.66 ± 0.55
	Conductivity (mS cm^−1^)	0.05 ± 0.02	1.16 ± 0.21	3.90 ± 0.81	8.42 ± 0.94	18.40 ± 2.06
W27B	Temperature (°C)	19.50 ± 0.17	19.53 ± 0.18	19.45 ± 0.15	19.53 ± 0.13	19.45 ± 0.19
	pH	7.73 ± 0.23	7.63 ± 0.29	7.49 ± 0.30	7.52 ± 0.27	7.68 ± 0.34
	Conductivity (mS cm^−1^)	0.05 ± 0.03	1.15 ± 0.27	4.33 ± 0.41	9.32 ± 1.38	17.02 ± 1.27
W110	Temperature (°C)	19.31 ± 0.29	19.42 ± 0.24	19.42 ± 0.14	19.38 ± 0.18	19.52 ± 0.21
	pH	7.63 ± 0.48	7.43 ± 0.40	7.43 ± 0.3	7.45 ± 0.43	7.44 ± 0.34
	Conductivity (mS cm^−1^)	0.05 ± 0.03	1.14 ± 0.19	4.76 ± 0.77	8.93 ± 0.73	17.19 ± 1.16

### Taxonomic composition

**Figure 1:**
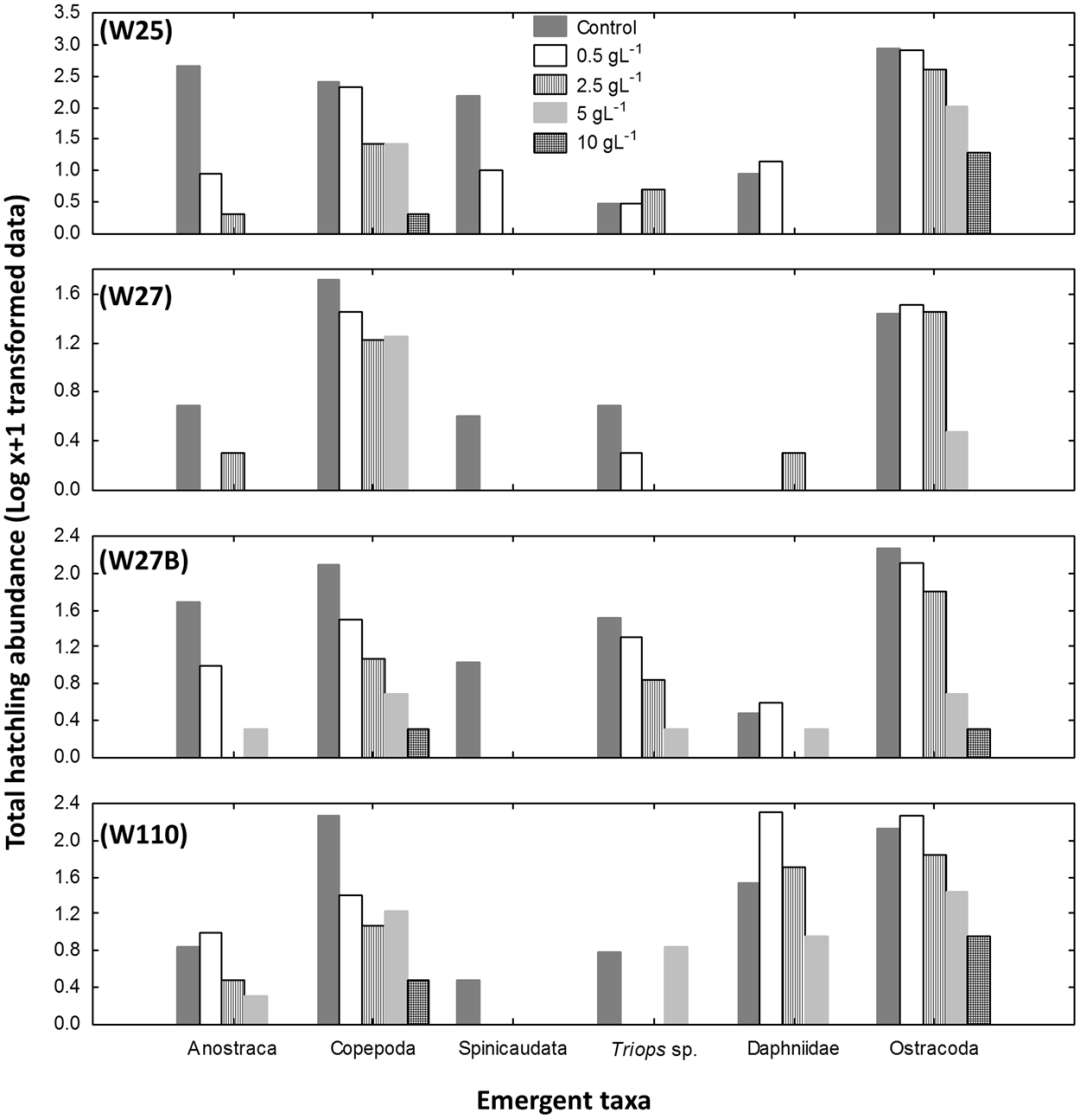
Total number of hatchlings (Log х + 1 transformed data) after 28 days of inundation of sediment from the four Eastern Cape Karoo wetlands at five salinity treatments.

**Figure 2:**
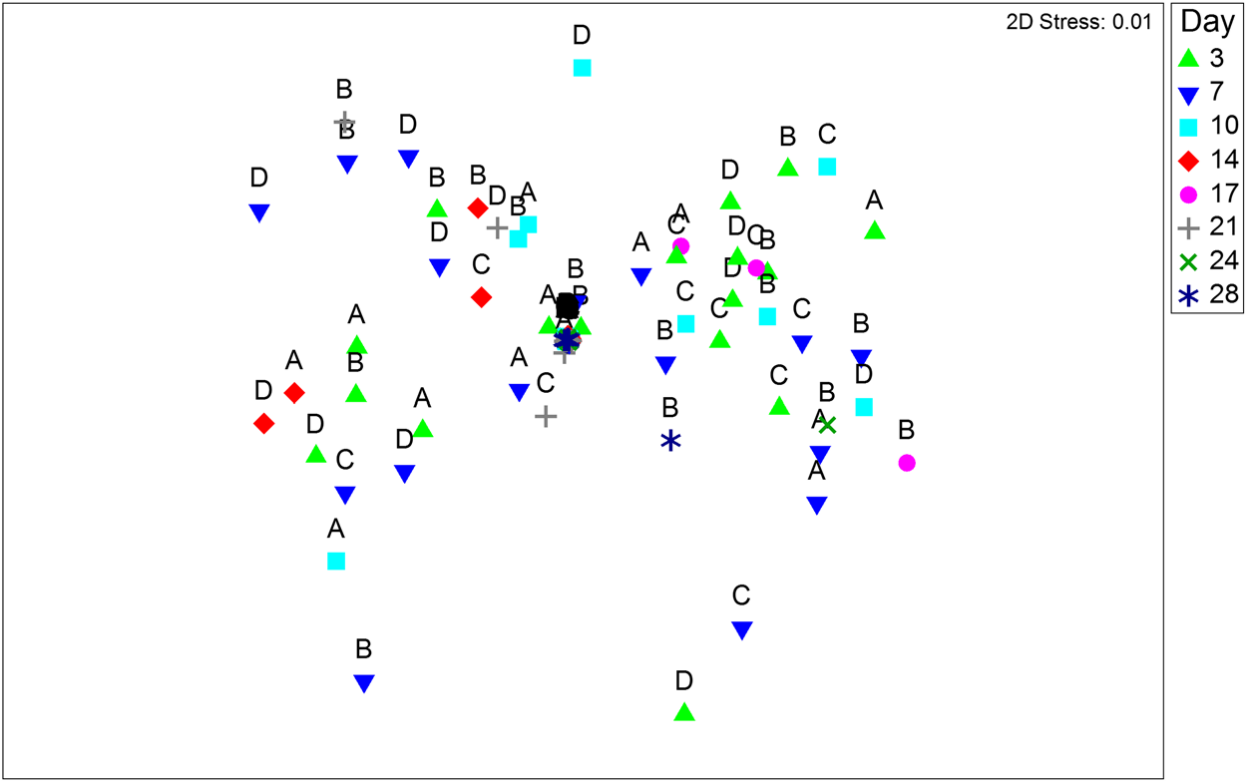
MDS ordination plot of emergent crustacean hatchlings based on the Bray-Curtis similarity among sites; W25 (A), W27 (B), W27B (C) and W110 (D). The data were pooled across all treatments per wetland.

Emergence of hatchlings from the sediments occurred over several days and was observed until the day of experiment termination. Crustaceans from six taxonomic orders (Table 2) emerged from the four depressional wetland sediments over the 28-day inundation period, see also Supplementary Table S2. Taxon richness was highest in the control and least in the 10 g L^−1^ treatment for all wetlands, but with high variability among wetlands (Table 2). Fairy shrimps (Anostraca), copepods (Copepoda), tadpole shrimps (Notostraca) and clam shrimps (Spinicaudata) were the first to emerge and were already collected on day 3 after inundation, while water fleas (Daphniidae) and seed shrimps (Ostracoda) were first collected only after day 7 (Supplementary Table S2). Salinities above 0.5 g L^−1^ reduced the proportion of Anostraca, Notostraca and Spinicaudata emergence to almost zero (see Supplementary Table S2), while Copepoda, Ostracoda and Daphniidae were the only taxa that emerged at the highest salinity level used (10 g L^−1^, Fig. 1). Ostracods were the most abundant in all depression wetlands, followed by copepods. However, anostracans were the most abundant on day 3 in the control sample for one of the depression wetlands (site W25). Results of the multi-dimensional scaling ordination (Fig. 2) showed no separation of sites indicating similar number of hatchlings. The large branchiopod taxa emergent from the sediments were of the genera  *Branchipodopsis*, *Cyzicus*, *Streptocephalus* and the notostracan species *Triops granarius* Lucas, 1864. More details on the common species that were recorded in the wetlands during the wet season sampling are given in Mabidi *et al*.^36^.

**Table 2:** Cumulative number of emergent hatchlings for each taxon and richness after 28 days of inundation of sediment from the Eastern Cape Karoo wetlands at five salinity treatments.

Wetland code	Taxa	Control	0.5 g L^−1^	2.5 g L^−1^	5 g L^−1^	10 g L^−1^
W25	Anostraca	473	8	1	0	0
	Copepoda	256	207	26	26	1
	Spinicaudata	157	9	0	0	0
	Notostraca	2	2	4	0	0
	Daphniidae	8	13	0	0	0
	Ostracoda	873	842	393	101	18
	Taxa richness	6	6	4	2	2
W27	Anostraca	4	0	1	0	0
	Copepoda	50	28	16	17	0
	Spinicaudata	3	0	0	0	0
	Notostraca	4	1	0	0	0
	Daphniidae	0	0	1	0	0
	Ostracoda	27	32	28	2	0
	Taxa richness	5	3	4	2	0
W27B	Anostraca	47	9	0	1	0
	Copepoda	125	31	11	4	1
	Spinicaudata	10	0	0	0	0
	Notostraca	32	19	6	1	0
	Daphniidae	2	3	0	4	1
	Ostracoda	187	129	63	4	1
	Taxa richness	6	5	3	5	2
W110	Anostraca	6	9	2	1	0
	Copepoda	182	24	11	16	2
	Spinicaudata	2	0	0	0	0
	Notostraca	5	0	0	6	0
	Daphniidae	33	201	50	8	0
	Ostracoda	135	180	69	26	8
	Taxa richness	6	4	4	5	2

### Hatchling abundance

**Figure 3:**
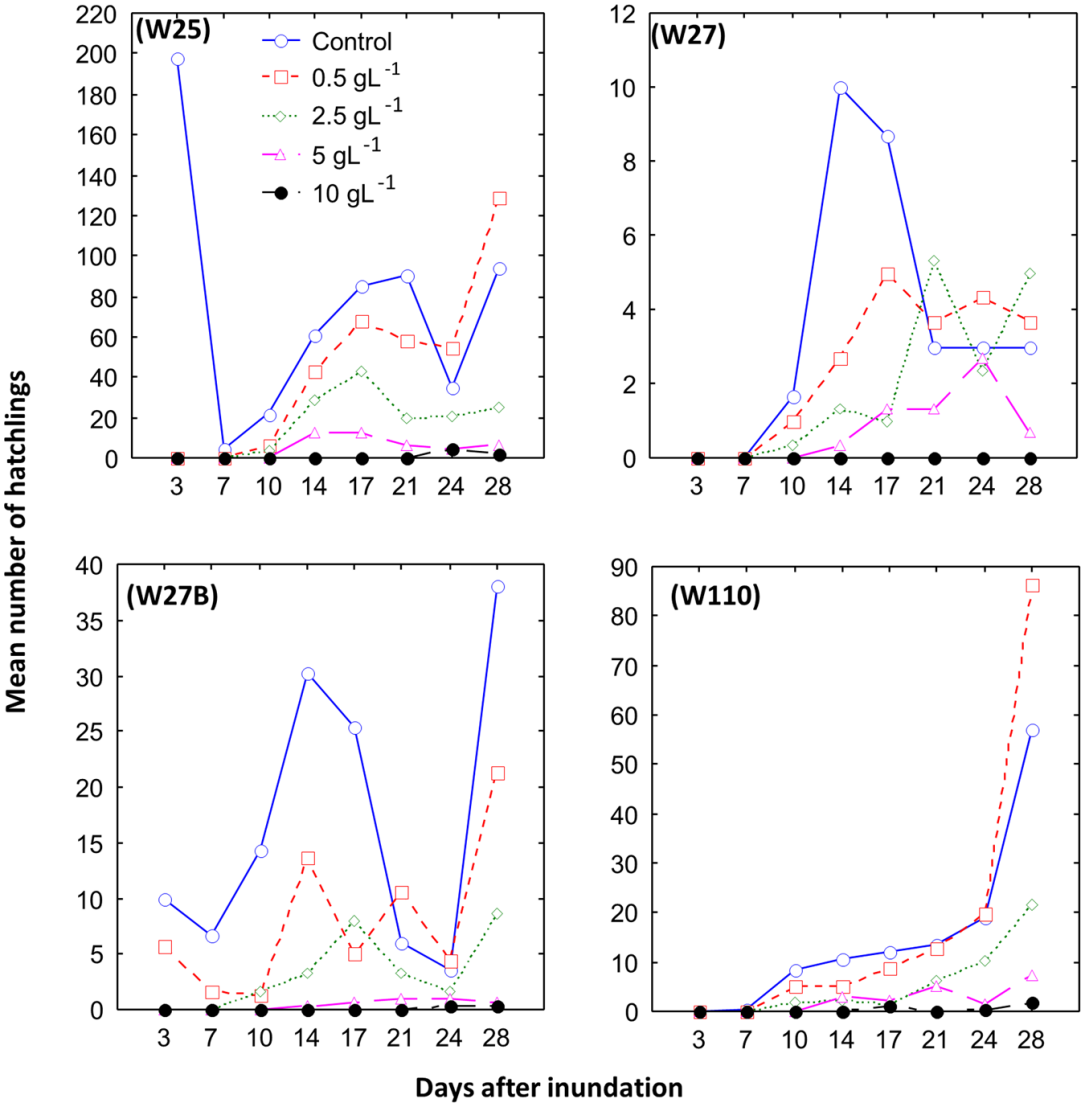
Number of hatchlings (mean, n = 3) emerged from sediments of the Eastern Cape Karoo wetlands at five salinity treatments.

The highest mean number of hatchlings was on day 3 in the control aquaria for depression wetland W25 (197.33), day 14 for W27 (10) and day 28 for W27B (38) (Fig. 3). However, for depression wetland W110, the highest mean number of hatchlings (86.33) was in the 0.5 gL^−1^ salinity treatment on day 28 (Fig. 3). No hatchlings were detected throughout the experiment at 10 g L^−1^ for sediments from wetland W27 (Fig. 3). The salinity treatment had a significant effect on hatchling abundance (F_4,40 = _103.95, P < 0.0001), as did the site from which wetland sediment was taken (F_3,40_ = 48.22, P < 0.0001, Table 3a). However, there was no significant interaction between these two factors (F_12,40_ = 1.61, P = 0.1261), indicating that the salinity effect was relatively consistent across sites. *Post hoc* tests revealed significant pairwise differences at site W25 for the control vs 5 g L^−1^ comparison, as well as for control vs 10 g L^−1^, 0.5 g L^−1^ vs 5 g L^−1^, 0.5 g L^−1^ vs 10 g L^−1^ and 2.5 g L^−1^ vs 10 g L^−1^ (Table 3b). At site W27, significant differences were reported for control vs 10 g L^−1^, 0.5 g L^−1^ vs 10 g L^−1^ and 2.5 g L^−1^ vs 10 g L^−1^. At site W27B, significant differences were reported for the control vs 5 g L^−1^, control vs 10 g L^−1^, 0.5 g L^−1^ vs 5 g L^−1^, 0.5 g L^−1^ vs 10 g L^−1^ and 2.5 g L^−1^ vs 10 g L^−1^, whilst at W110 significant differences were found for control vs 10 g L^−1^, 0.5 g L^−1^ vs 10 g L^−1^ and 2.5 g L^−1^ vs 10 g L^−1^ (Table 3b).

**Table 3:** Results of the two-way ANOVA comparing hatchling abundance across the different salinity treatments and sites (a).

(a)	SS	df	MS	F	P
Salinity	23.3325	4	5.8331	103.953	<0.0001
Site	8.1174	3	2.7058	48.221	<0.0001
Salinity x site	1.0881	12	0.0907	1.616	0.1261
Residual	2.2445	40	0.0561		
**(b)**	**Site**	**Significant comparisons**			**P value**
	W25	Control vs 5 g L^−1^			0.000187
		Control vs 10 g L^−1^			0.000179
		0.5 g L^−1^ vs 5 g L^−1^			0.000586
		0.5 g L^−1^ vs 10 g L^−1^			0.000179
		2.5 g L^−1^ vs 10 g L^−1^			0.000181
	W27	Control vs 10 g L^−1^			0.000179
		0.5 g L^−1^ vs 10 g L^−1^			0.000182
		2.5 g L^−1^ vs 10 g L^−1^			0.000203
	W27B	Control vs 5 g L^−1^			0.000179
		Control vs 10 g L^−1^			0.000179
		0.5 g L^−1^ vs 5 g L^−1^			0.000205
		0.5 g L^−1^ vs 10 g L^−1^			0.000179
		2.5 g L^−1^ vs 10 g L^−1^			0.000297
	W110	Control vs 10 g L^−1^			0.000180
		0.5 g L^−1^ vs 10 g L^−1^			0.000179
		2.5 g L^−1^ vs 10 g L^−1^			0.001076

Significant results for the pairwise comparisons of treatment levels for each site are also presented (b).

## Discussion

Anthropogenic or secondary salinisation of freshwaters is a growing concern throughout the world^3^. It results from a range of sources including agriculture, mining and urban development^2^. Most freshwater biota do not tolerate large increases in salt concentrations^17^ and salinity has been reported to be the most important factor influencing the distribution of branchiopods^37^. Our results show that hatchling abundance of crustaceans was sharply reduced at salinities of 2.5 g L^−1^ and above across all the wetlands. This reduced emergence of hatchlings at 2.5 g L^−1^ and above can be attributed to increased salinity blocking the required cues to trigger emergence^24^ or toxicity of salt which can cause death soon after emergence^3,34^. It has been reported that high concentrations of salt increase the osmotic pressure of the water outside the egg^38^, thus more glycerol must be produced by the metanauplius^38^ to enable the movement of sufficient quantities of water into the egg and burst the outer membrane. Consequently, the metanauplius depletes its energy reserves in the process^39^, resulting in reduced viability.

Reduced hatching abundances of freshwater branchiopods in response to an increase in salinity are reported for various species of Anostraca^40^. For instance, the hatching of the fairy shrimp *Streptocephalus dichotomus* Baird, was inhibited by elevated conductivities^41^, while low conductivities promoted hatching of the fairy shrimp *Branchipodopsis wolfi* Daday^27^. Similarly, no hatching of the *Branchipodopsis* fairy shrimp (Anostraca) under elevated conductivities was reported in another study^24^. This was attributed to a mechanism aimed at avoiding abortive hatching, suggesting that low initial conductivities can be predictors for the length of inundations. Similarly, our results showed that reduction in emergence was pronounced particularly for the Anostraca, indicating the adaptive value of low conductivity as a hatching cue for crustaceans in temporary wetlands^24^. Many other studies have reported loss of invertebrate diversity as salinity increases^3,4,16,42^. Our results show that ostracods, copepods and cladocerans were the only taxa that emerged from sediments inundated with solutions exhibiting 10 g L^−1^ salinity. Ostracods are known to occupy habitats ranging from fresh to highly saline conditions, while copepods have also been recorded in a saline pan in Mpumalanga, South Africa^43^. Other researchers that have investigated the distribution of cladocerans in different waterbodies along salinity gradients have reported tolerance of increased salinity^44–46^. Some cladocerans species have been found to be tolerant to field salinities above a 15 mS cm^−1^ threshold^47^.

Depression wetlands are regarded as good early warning systems for biological impacts of shifting climate^47,48^ and crustaceans have the potential to be used as bio-indicators of environmental change^49^. The results of this study indicate that increasing salinity can drastically inhibit emergence of hatchlings from egg bank sediments of depression wetlands from the Eastern Cape Karoo region. Therefore, the possible salinization of Eastern Cape Karoo depression wetlands because of hydraulic fracturing or climate change can result in loss of sensitive taxa such as Anostraca, Notostraca and Spinicaudata. This will consequently enable the proliferation of less sensitive taxa such as Copepoda and Ostracoda, resulting in loss of biodiversity and changes in community composition^32^. These changes may affect nutrient cycling, other organisms in the food web and disrupt normal ecosystem functioning. For example, *Triops* (Notostraca) has been reported to be a keystone species in temporary wetlands and significantly influences ecosystem functioning by triggering changes in the composition of temporary wetland communities and affects water quality through bioturbation^50^. Several studies have shown that increased salinity levels are associated with lower invertebrate diversity and decreased taxon abundance^29,51,52^ and we have reported similar findings in this study. Thus, any deleterious effects of increased salinity are likely to affect broader ecosystem processes such as primary productivity, decomposition, nutrient recycling and energy and material flow through trophic webs^53^. Therefore, even though the copepods and ostracods may be tolerant of salination in the studied systems, the loss of Notostraca may induce vulnerability because of flow-on effects from the species or taxonomic group’s intolerance^34,53^ thereby reducing the resilience of the wetland ecosystems^54^. It is therefore important that communities are diverse to account for the interdependence of different species within communities^53^. Depression wetlands are the most at risk of pollution because of their small volume and shallow depth^55^. These systems often have no outlet, and this has a potential to increase the concentration of toxicants. Natural depression wetlands contribute immensely to the variability in aquatic habitats and biodiversity in the Eastern Cape Karoo region^55^. Thus, salinisation of these systems may lead to considerable decline in invertebrate diversity in the region, with cascading effects on food webs and ecosystem functions. However, there is limited information available on the impact of salinity on trophic interactions^52^.

The region is already experiencing water scarcity (as caused by climate change) and the imminent shale gas development in the region has a potential to increase water demand and exacerbate the problem of freshwater salinisation. Therefore, further studies on the relationship between increasing salinity and the ecology of temporary wetland inhabitants are necessary in the region. This information is needed to estimate sensitivity of depression wetland biota to environmental change, so that proper conservation measures can be applied. Furthermore, knowledge of the tolerance levels of the biota in these systems will help predict scenarios for future environmental change. This will enable the formulation of adequate legislation, and suitable monitoring strategies to be employed prior to shale gas exploration.

## Methods

### Study area

Sediment samples containing dormant egg banks were collected in November 2016 from four depression wetlands (W25, W27, W27B and W110) occurring within the area of the Eastern Cape Karoo demarcated for shale gas drilling (Table 1). These wetlands were specifically selected to maximise the chance of collecting sediment with resting eggs, because they were found to contain large branchiopod species from more than four orders during collections made in the previous wet season^36^. The study area falls within the semi-arid Nama Karoo Biome. A more detailed description of the study area and sampling sites is given in Mabidi, *et al*.^35^.

### Sediment collection

A hand-held shovel was used to collect sediment samples from the upper 3 cm of sediment at twenty locations spread throughout the wetland basin, with most of the collecting effort being focused on collecting the twenty sediment samples in the deepest section of each wetland. As these are the last to dry up, the sediment contains more viable newly deposited egg banks than anywhere else in the wetland^39,49^. The samples were transported back to the laboratory where they were gently broken up by hand to avoid damaging the eggs and mixed to form a composite sample for each wetland. A composite sample would maximise the number of zooplankton propagules and minimise the variability within each wetland. All samples were stored at room temperature in the laboratory until the hatching experiment commenced in January 2017.

### Hatching experiment

Propagule hatching was conducted at constant fluorescent lighting (24 hr) at 18 °C in a Daihan Labtech Programmable Growth Chamber (Model LGC–4203). These conditions have been previously shown to be ideal for hatching the Karoo resting eggs (Mabidi *et al*. unpublished. data). To check for effects of salinity on hatching rate, sediment samples were cultured in 2056.28 cm^3^ aquaria with different salt concentrations. Each dry sediment sample weighing 70 g was inundated with 2 L of distilled water (control) or one of the following four salinity treatments: 0.5 g L^−1^, 2.5 g L^−1^, 5 g L^−1^, and 10 g L^−1^. The treatments were created by adding the appropriate amount of commercial aquarium salt (Blue Treasure SPS sea salt) per Litre of distilled water and the salinity measurement verified by a salinity meter (Hanna Multiparameter Meter (Model Hi 7609829). Three replicates were used for the control and for all the four treatments as well (i.e. 210 g of sediment for the control and each treatment), which amounted to a total of 1050 g sediment per each wetland. Thus, 15 samples (each weighing 70 g) were used per each wetland. The highest salinity treatment was set at 10 g L^−1^ because this was the highest value measured from ground water (boreholes) in the study area (Divan Stroebel, pers. comm.). All aquaria were sampled twice per week during a period of 28 days after rehydration. Prior to each sample of invertebrates being taken, key physico-chemical variables (pH, conductivity, temperature) were measured in each aquarium using a Hanna Multiparameter Meter (Model Hi 7609829). The medium in each aquarium was carefully decanted and filtered over a 100 µm sieve, and the filtrate poured back into the container. Additional distilled water was only added when necessary to keep the containers from drying by evaporation. Hatchlings retained on the sieve were fixed in 5% formalin and stained with Rose Bengal solution, counted and identified to order level under a Zeiss stereo microscope (80X magnification) using identification guides by Day *et al*.^43,56^. Even though occasional turbellarians hatched from some of the sediments, their numbers were very low, thus abundances were only determined for crustaceans (i.e. anostracans, cladocerans, copepods notostracans, ostracods and spinicaudatans).

### Data analysis

Differences in hatchling abundances among the five salinity treatment levels (fixed factor: ‘salinity’ with five levels: control; 0.5 g L^−1^; 2.5 g L^−1^; 5 g L^−1^; and 10 g L^−1^) were tested using ANOVA. The four wetlands were included as a random factor (‘sites’) in a two-way crossed design, given that we were interested in whether the treatment effect varied spatially across wetlands from different areas of the Karoo. For each of the three replicate containers per treatment at each site, the hatchling counts from the eight repeated samples were summed and this summed abundance was used in the analysis. Given the initially skewed distribution of the response variable representing abundance, the data were log (x + 1) transformed and this resulted in a distribution that closely approximated a Gaussian curve (Shapiro-Wilk test). Plots of the residuals versus group means indicated similar variances among groups and there was no indication of a mean-variance relationship, hence the core assumptions for parametric ANOVA were upheld. *Post hoc* Tukey tests were used to investigate pairwise comparisons, using a Bonferroni-adjusted significance level of 0.00125 for the *post hoc* tests, which was sequentially adjusted following the procedure of Holm^57^. Non-metric multidimensional scaling (MDS) ordination was used to visualise temporal differences in assemblage composition (i.e. between successive samplings), as well as spatial differences (i.e. among sites). The data were pooled across all treatments per wetland. The MDS was derived from a Bray-Curtis similarity matrix representing assemblage composition (abundance data), with the addition of a dummy variable to cope with the presence of double-zeros. Univariate tests were carried out using the software package Statistica 10 (Stat Soft, Inc., Tulsa, Oklahoma, USA). The MDS ordination was performed using PRIMER v6 software^58^. An *a priori* significance level of α = 0.05 was used for all statistical tests, although this was Bonferroni-adjusted for the *post hoc* Tukey tests.

### Data Availability

All data generated or analysed during this study are included in this manuscript (and its Supplementary Information files).

### Ethics statement

Permission for fieldwork and scientific collection of sediment in depression wetlands of the semi-arid Eastern Cape Karoo region earmarked for shale gas exploration was granted by the Eastern Cape Department of Economic Affairs, Environmental Affairs and Tourism (Cacadu Region.). The research involved the collection of sediment and handling of invertebrates. The methods were carried out in accordance with the relevant guidelines and regulations, as approved in terms of animal care and use procedures by the Nelson Mandela University Ethics Committee (ethics clearance reference number is A14-SCI-ZOO-010**)**.

## Electronic supplementary material

Supplementary Information
