# Supplementary material for: Increasing salinity drastically reduces hatching success of crustaceans from depression wetlands of the semi-arid Eastern Cape Karoo region, South Africa
Source: Sci Rep. 2018 Apr 13;8:5983. doi: 10.1038/s41598-018-24137-0 (PMC5899145; doi:10.1038/s41598-018-24137-0)
Supplement: Supplementary file 1 — Supplementary Information [file 41598_2018_24137_MOESM1_ESM.docx]

**Increasing salinity drastically reduces hatching success of crustaceans from depression wetlands of the semi-arid Eastern Cape Karoo region, South Africa**

Annah Mabidi^1,2*^ Matthew S. Bird^1,3^ and Renzo Perissinotto^1^

^1^South African Research Chairs Initiative, Shallow Water Ecosystems, Nelson Mandela University, PO Box 77000, Port Elizabeth 6031, South Africa.

^2^Africa Earth Observatory Network, Nelson Mandela University, PO Box 77000, Port Elizabeth, 6031 South Africa.

^3^Department of Zoology, University of Johannesburg, P.O. Box, 524, Auckland Park, Johannesburg, 2006 South Africa.

^*^Corresponding author. Email: [annahanusa@gmail.com](mailto:annahanusa@gmail.com)

| **Wetland Code** | **Latitude** | **Longitude** | **Altitude (m.a.s.l)** | **Temperature (°C)** | **Conductivity (mS cm^-1^)** | **pH** |
| --- | --- | --- | --- | --- | --- | --- |
| W25 | −32.603381 | 24.651856 | 598 | 25.8; 27.8 | 0.07; 0.08 | 8.45; 8.68 |
| W27 | −32.494572 | 24.299996 | 661 | 18.2; 22.5 | 0.19; 0.19 | 7.90; 8.28 |
| W27B | −32.507438 | 24.655214 | 654 | 20.0; 29.3 | 0.08; 0.08 | 7.46; 8.53 |
| W110 | -32.626712 | 24.192674 | 725 | 18.7; 23.2 | 0.16; 0.22 | 7.65; 8.74 |

**Table S1**. **Location (latitude and longitude in decimal points) of sampling sites in the study area.** The mean values of key variables measured during 2014 / 2015 wet season sampling in November 2014 and April 2015 (months with maximum precipitation) are also given.

**Increasing salinity drastically reduces hatching success of crustaceans from depression wetlands of the semi-arid Eastern Cape Karoo region, South Africa**

Annah Mabidi^1,2*^ Matthew S. Bird^1,3^ and Renzo Perissinotto^1^

^1^South African Research Chairs Initiative, Shallow Water Ecosystems, Nelson Mandela University, PO Box 77000, Port Elizabeth 6031, South Africa.

^2^Africa Earth Observatory Network, Nelson Mandela University, PO Box 77000, Port Elizabeth, 6031 South Africa.

^3^Department of Zoology, University of Johannesburg, P.O. Box, 524, Auckland Park, Johannesburg, 2006 South Africa.

^*^Corresponding author. Email: [annahanusa@gmail.com](mailto:annahanusa@gmail.com)

| Code | Day | Taxa | Control |  |  | 0.5 g L^−1^ |  |  | 2.5 g L^−1^ |  |  | 5 g L^−1^ |  |  | 10 g L^−1^ |  |  |
| --- | --- | --- | --- | --- | --- | --- | --- | --- | --- | --- | --- | --- | --- | --- | --- | --- | --- |
|  |  |  | Rep 1 | Rep 2 | Rep 3 | Rep 1 | Rep 2 | Rep 3 | Rep 1 | Rep 2 | Rep 3 | Rep 1 | Rep 2 | Rep 3 | Rep 1 | Rep 2 | Rep 3 |
| W25 | 3 | Anostraca | 136 | 102 | 211 | 0 | 0 | 0 | 0 | 0 | 0 | 0 | 0 | 0 | 0 | 0 | 0 |
|  | 7 | Anostraca | 2 | 0 | 0 | 0 | 0 | 0 | 0 | 0 | 0 | 0 | 0 | 0 | 0 | 0 | 0 |
|  | 10 | Anostraca | 0 | 8 | 0 | 0 | 7 | 0 | 1 | 0 | 0 | 0 | 0 | 0 | 0 | 0 | 0 |
|  | 14 | Anostraca | 3 | 5 | 1 | 1 | 0 | 0 | 0 | 0 | 0 | 0 | 0 | 0 | 0 | 0 | 0 |
|  | 17 | Anostraca | 1 | 0 | 4 | 0 | 0 | 0 | 0 | 0 | 0 | 0 | 0 | 0 | 0 | 0 | 0 |
|  | 21 | Anostraca | 0 | 0 | 0 | 0 | 0 | 0 | 0 | 0 | 0 | 0 | 0 | 0 | 0 | 0 | 0 |
|  | 24 | Anostraca | 0 | 0 | 0 | 0 | 0 | 0 | 0 | 0 | 0 | 0 | 0 | 0 | 0 | 0 | 0 |
|  | 28 | Anostraca | 0 | 0 | 0 | 0 | 0 | 0 | 0 | 0 | 0 | 0 | 0 | 0 | 0 | 0 | 0 |
|  |  |  |  |  |  |  |  |  |  |  |  |  |  |  |  |  |  |
|  | 3 | Copepoda | 0 | 0 | 0 | 0 | 0 | 0 | 0 | 0 | 0 | 0 | 0 | 0 | 0 | 0 | 0 |
|  | 7 | Copepoda | 1 | 0 | 0 | 0 | 0 | 0 | 0 | 0 | 0 | 0 | 0 | 0 | 0 | 0 | 0 |
|  | 10 | Copepoda | 0 | 52 | 0 | 2 | 6 | 0 | 9 | 0 | 0 | 0 | 0 | 0 | 0 | 0 | 0 |
| Code | Day | Taxa | Control |  |  | 0.5 g L^−1^ |  |  | 2.5 g L^−1^ |  |  | 5 g L^−1^ |  |  | 10 g L^−1^ |  |  |
|  |  |  | Rep 1 | Rep 2 | Rep 3 | Rep 1 | Rep 2 | Rep 3 | Rep 1 | Rep 2 | Rep 3 | Rep 1 | Rep 2 | Rep 3 | Rep 1 | Rep 2 | Rep 3 |
|  | 14 | Copepoda | 2 | 13 | 3 | 23 | 0 | 3 | 3 | 0 | 0 | 0 | 0 | 11 | 0 | 0 | 0 |
|  | 17 | Copepoda | 27 | 33 | 16 | 24 | 39 | 15 | 0 | 1 | 5 | 2 | 1 | 3 | 0 | 0 | 0 |
|  | 21 | Copepoda | 5 | 25 | 14 | 4 | 7 | 17 | 2 | 0 | 0 | 1 | 7 | 1 | 0 | 0 | 0 |
|  | 24 | Copepoda | 9 | 13 | 11 | 4 | 16 | 16 | 4 | 0 | 1 | 0 | 0 | 0 | 1 | 0 | 0 |
|  | 28 | Copepoda | 15 | 4 | 13 | 7 | 20 | 4 | 1 | 0 | 0 | 0 | 0 | 0 | 0 | 0 | 0 |
|  |  |  |  |  |  |  |  |  |  |  |  |  |  |  |  |  |  |
|  | 3 | Spinicaudata | 53 | 54 | 36 | 0 | 0 | 0 | 0 | 0 | 0 | 0 | 0 | 0 | 0 | 0 | 0 |
|  | 7 | Spinicaudata | 8 | 0 | 0 | 0 | 0 | 0 | 0 | 0 | 0 | 0 | 0 | 0 | 0 | 0 | 0 |
|  | 10 | Spinicaudata | 0 | 2 | 3 | 0 | 5 | 0 | 0 | 0 | 0 | 0 | 0 | 0 | 0 | 0 | 0 |
|  | 14 | Spinicaudata | 0 | 0 | 0 | 0 | 0 | 0 | 0 | 0 | 0 | 0 | 0 | 0 | 0 | 0 | 0 |
|  | 17 | Spinicaudata | 0 | 1 | 0 | 0 | 0 | 0 | 0 | 0 | 0 | 0 | 0 | 0 | 0 | 0 | 0 |
|  | 21 | Spinicaudata | 0 | 0 | 0 | 0 | 2 | 1 | 0 | 0 | 0 | 0 | 0 | 0 | 0 | 0 | 0 |
|  | 24 | Spinicaudata | 0 | 0 | 0 | 0 | 1 | 0 | 0 | 0 | 0 | 0 | 0 | 0 | 0 | 0 | 0 |
|  | 28 | Spinicaudata | 0 | 0 | 0 | 0 | 0 | 0 | 0 | 0 | 0 | 0 | 0 | 0 | 0 | 0 | 0 |
|  |  |  |  |  |  |  |  |  |  |  |  |  |  |  |  |  |  |
|  | 3 | Notostraca | 0 | 0 | 0 | 0 | 0 | 0 | 0 | 0 | 0 | 0 | 0 | 0 | 0 | 0 | 0 |
|  | 7 | Notostraca | 2 | 0 | 0 | 0 | 0 | 0 | 0 | 0 | 0 | 0 | 0 | 0 | 0 | 0 | 0 |
|  | 10 | Notostraca | 0 | 0 | 0 | 0 | 0 | 0 | 0 | 0 | 0 | 0 | 0 | 0 | 0 | 0 | 0 |
|  | 14 | Notostraca | 0 | 0 | 0 | 2 | 0 | 0 | 4 | 0 | 0 | 0 | 0 | 0 | 0 | 0 | 0 |
|  | 17 | Notostraca | 0 | 0 | 0 | 0 | 0 | 0 | 0 | 0 | 0 | 0 | 0 | 0 | 0 | 0 | 0 |
| Code | Day | Taxa | Control |  |  | 0.5 g L^−1^ |  |  | 2.5 g L^−1^ |  |  | 5 g L^−1^ |  |  | 10 g L^−1^ |  |  |
|  |  |  | Rep 1 | Rep 2 | Rep 3 | Rep 1 | Rep 2 | Rep 3 | Rep 1 | Rep 2 | Rep 3 | Rep 1 | Rep 2 | Rep 3 | Rep 1 | Rep 2 | Rep 3 |
|  | 21 | Notostraca | 0 | 0 | 0 | 0 | 0 | 0 | 0 | 0 | 0 | 0 | 0 | 0 | 0 | 0 | 0 |
|  | 24 | Notostraca | 0 | 0 | 0 | 0 | 0 | 0 | 0 | 0 | 0 | 0 | 0 | 0 | 0 | 0 | 0 |
|  | 28 | Notostraca | 0 | 0 | 0 | 0 | 0 | 0 | 0 | 0 | 0 | 0 | 0 | 0 | 0 | 0 | 0 |
|  |  |  |  |  |  |  |  |  |  |  |  |  |  |  |  |  |  |
|  | 3 | Daphniidae | 0 | 0 | 0 | 0 | 0 | 0 | 0 | 0 | 0 | 0 | 0 | 0 | 0 | 0 | 0 |
|  | 7 | Daphniidae | 0 | 0 | 0 | 0 | 0 | 0 | 0 | 0 | 0 | 0 | 0 | 0 | 0 | 0 | 0 |
|  | 10 | Daphniidae | 0 | 0 | 0 | 0 | 0 | 0 | 0 | 0 | 0 | 0 | 0 | 0 | 0 | 0 | 0 |
|  | 14 | Daphniidae | 3 | 1 | 4 | 1 | 9 | 3 | 0 | 0 | 0 | 0 | 0 | 0 | 0 | 0 | 0 |
|  | 17 | Daphniidae | 0 | 0 | 0 | 0 | 0 | 0 | 0 | 0 | 0 | 0 | 0 | 0 | 0 | 0 | 0 |
|  | 21 | Daphniidae | 0 | 0 | 0 | 0 | 0 | 0 | 0 | 0 | 0 | 0 | 0 | 0 | 0 | 0 | 0 |
|  | 24 | Daphniidae | 0 | 0 | 0 | 0 | 0 | 0 | 0 | 0 | 0 | 0 | 0 | 0 | 0 | 0 | 0 |
|  | 28 | Daphniidae | 0 | 0 | 0 | 0 | 0 | 0 | 0 | 0 | 0 | 0 | 0 | 0 | 0 | 0 | 0 |
|  |  |  |  |  |  |  |  |  |  |  |  |  |  |  |  |  |  |
|  | 3 | Ostracoda | 0 | 0 | 0 | 0 | 0 | 0 | 0 | 0 | 0 | 0 | 0 | 0 | 0 | 0 | 0 |
|  | 7 | Ostracoda | 0 | 0 | 0 | 0 | 0 | 0 | 0 | 0 | 0 | 0 | 0 | 0 | 0 | 0 | 0 |
|  | 10 | Ostracoda | 0 | 1 | 0 | 0 | 0 | 0 | 0 | 0 | 0 | 0 | 0 | 0 | 0 | 0 | 0 |
|  | 14 | Ostracoda | 30 | 53 | 65 | 24 | 31 | 33 | 47 | 21 | 11 | 23 | 1 | 3 | 0 | 0 | 0 |
|  | 17 | Ostracoda | 75 | 64 | 34 | 51 | 41 | 35 | 22 | 49 | 52 | 30 | 2 | 0 | 0 | 0 | 0 |
|  | 21 | Ostracoda | 41 | 107 | 80 | 62 | 54 | 28 | 18 | 34 | 6 | 7 | 1 | 1 | 0 | 0 | 0 |
|  | 24 | Ostracoda | 29 | 22 | 22 | 44 | 7 | 75 | 8 | 37 | 13 | 13 | 0 | 1 | 5 | 7 | 1 |
| Code | Day | Taxa | Control |  |  | 0.5 g L^−1^ |  |  | 2.5 g L^−1^ |  |  | 5 g L^−1^ |  |  | 10 g L^−1^ |  |  |
|  |  |  | Rep 1 | Rep 2 | Rep 3 | Rep 1 | Rep 2 | Rep 3 | Rep 1 | Rep 2 | Rep 3 | Rep 1 | Rep 2 | Rep 3 | Rep 1 | Rep 2 | Rep 3 |
|  | 28 | Ostracoda | 220 | 21 | 9 | 175 | 108 | 74 | 33 | 34 | 8 | 14 | 1 | 4 | 4 | 0 | 1 |
|  |  |  |  |  |  |  |  |  |  |  |  |  |  |  |  |  |  |
| W27 | 3 | Anostraca | 0 | 0 | 0 | 0 | 0 | 0 | 0 | 0 | 0 | 0 | 0 | 0 | 0 | 0 | 0 |
|  | 7 | Anostraca | 0 | 0 | 0 | 0 | 0 | 0 | 0 | 0 | 0 | 0 | 0 | 0 | 0 | 0 | 0 |
|  | 10 | Anostraca | 0 | 0 | 0 | 0 | 0 | 0 | 0 | 0 | 0 | 0 | 0 | 0 | 0 | 0 | 0 |
|  | 14 | Anostraca | 2 | 0 | 1 | 0 | 0 | 0 | 0 | 1 | 0 | 0 | 0 | 0 | 0 | 0 | 0 |
|  | 17 | Anostraca | 0 | 1 | 0 | 0 | 0 | 0 | 0 | 0 | 0 | 0 | 0 | 0 | 0 | 0 | 0 |
|  | 21 | Anostraca | 0 | 0 | 0 | 0 | 0 | 0 | 0 | 0 | 0 | 0 | 0 | 0 | 0 | 0 | 0 |
|  | 24 | Anostraca | 0 | 0 | 0 | 0 | 0 | 0 | 0 | 0 | 0 | 0 | 0 | 0 | 0 | 0 | 0 |
|  | 28 | Anostraca | 0 | 0 | 0 | 0 | 0 | 0 | 0 | 0 | 0 | 0 | 0 | 0 | 0 | 0 | 0 |
|  |  |  |  |  |  |  |  |  |  |  |  |  |  |  |  |  |  |
|  | 3 | Copepoda | 0 | 0 | 0 | 0 | 0 | 0 | 0 | 0 | 0 | 0 | 0 | 0 | 0 | 0 | 0 |
|  | 7 | Copepoda | 0 | 0 | 0 | 0 | 0 | 0 | 0 | 0 | 0 | 0 | 0 | 0 | 0 | 0 | 0 |
|  | 10 | Copepoda | 0 | 0 | 0 | 0 | 0 | 0 | 0 | 0 | 0 | 0 | 0 | 0 | 0 | 0 | 0 |
|  | 14 | Copepoda | 12 | 10 | 2 | 1 | 3 | 0 | 1 | 0 | 0 | 0 | 0 | 1 | 0 | 0 | 0 |
|  | 17 | Copepoda | 11 | 7 | 3 | 5 | 1 | 1 | 2 | 0 | 0 | 3 | 0 | 1 | 0 | 0 | 0 |
|  | 21 | Copepoda | 0 | 0 | 0 | 0 | 6 | 0 | 3 | 3 | 0 | 1 | 2 | 1 | 0 | 0 | 0 |
|  | 24 | Copepoda | 0 | 1 | 1 | 3 | 2 | 3 | 0 | 0 | 0 | 3 | 4 | 0 | 0 | 0 | 0 |
|  | 28 | Copepoda | 0 | 0 | 3 | 0 | 3 | 0 | 3 | 2 | 2 | 0 | 1 | 0 | 0 | 0 | 0 |
|  |  |  |  |  |  |  |  |  |  |  |  |  |  |  |  |  |  |
| Code | Day | Taxa | Control |  |  | 0.5 g L^−1^ |  |  | 2.5 g L^−1^ |  |  | 5 g L^−1^ |  |  | 10 g L^−1^ |  |  |
|  |  |  | Rep 1 | Rep 2 | Rep 3 | Rep 1 | Rep 2 | Rep 3 | Rep 1 | Rep 2 | Rep 3 | Rep 1 | Rep 2 | Rep 3 | Rep 1 | Rep 2 | Rep 3 |
|  | 3 | Spinicaudata | 0 | 0 | 0 | 0 | 0 | 0 | 0 | 0 | 0 | 0 | 0 | 0 | 0 | 0 | 0 |
|  | 7 | Spinicaudata | 0 | 0 | 0 | 0 | 0 | 0 | 0 | 0 | 0 | 0 | 0 | 0 | 0 | 0 | 0 |
|  | 10 | Spinicaudata | 0 | 0 | 0 | 0 | 0 | 0 | 0 | 0 | 0 | 0 | 0 | 0 | 0 | 0 | 0 |
|  | 14 | Spinicaudata | 0 | 0 | 0 | 0 | 0 | 0 | 0 | 0 | 0 | 0 | 0 | 0 | 0 | 0 | 0 |
|  | 17 | Spinicaudata | 0 | 0 | 0 | 0 | 0 | 0 | 0 | 0 | 0 | 0 | 0 | 0 | 0 | 0 | 0 |
|  | 21 | Spinicaudata | 1 | 0 | 0 | 0 | 0 | 0 | 0 | 0 | 0 | 0 | 0 | 0 | 0 | 0 | 0 |
|  | 24 | Spinicaudata | 1 | 0 | 0 | 0 | 0 | 0 | 0 | 0 | 0 | 0 | 0 | 0 | 0 | 0 | 0 |
|  | 28 | Spinicaudata | 1 | 0 | 0 | 0 | 0 | 0 | 0 | 0 | 0 | 0 | 0 | 0 | 0 | 0 | 0 |
|  |  |  |  |  |  |  |  |  |  |  |  |  |  |  |  |  |  |
|  | 3 | Notostraca | 0 | 0 | 0 | 0 | 0 | 0 | 0 | 0 | 0 | 0 | 0 | 0 | 0 | 0 | 0 |
|  | 7 | Notostraca | 0 | 0 | 0 | 0 | 0 | 0 | 0 | 0 | 0 | 0 | 0 | 0 | 0 | 0 | 0 |
|  | 10 | Notostraca | 0 | 0 | 4 | 0 | 1 | 0 | 0 | 0 | 0 | 0 | 0 | 0 | 0 | 0 | 0 |
|  | 14 | Notostraca | 0 | 0 | 0 | 0 | 0 | 0 | 0 | 0 | 0 | 0 | 0 | 0 | 0 | 0 | 0 |
|  | 17 | Notostraca | 0 | 0 | 0 | 0 | 0 | 0 | 0 | 0 | 0 | 0 | 0 | 0 | 0 | 0 | 0 |
|  | 21 | Notostraca | 0 | 0 | 0 | 0 | 0 | 0 | 0 | 0 | 0 | 0 | 0 | 0 | 0 | 0 | 0 |
|  | 24 | Notostraca | 0 | 0 | 0 | 0 | 0 | 0 | 0 | 0 | 0 | 0 | 0 | 0 | 0 | 0 | 0 |
|  | 28 | Notostraca | 0 | 0 | 0 | 0 | 0 | 0 | 0 | 0 | 0 | 0 | 0 | 0 | 0 | 0 | 0 |
|  |  |  |  |  |  |  |  |  |  |  |  |  |  |  |  |  |  |
|  | 3 | Daphniidae | 0 | 0 | 0 | 0 | 0 | 0 | 0 | 0 | 0 | 0 | 0 | 0 | 0 | 0 | 0 |
|  | 7 | Daphniidae | 0 | 0 | 0 | 0 | 0 | 0 | 0 | 0 | 0 | 0 | 0 | 0 | 0 | 0 | 0 |
| Code | Day | Taxa | Control |  |  | 0.5 g L^−1^ |  |  | 2.5 g L^−1^ |  |  | 5 g L^−1^ |  |  | 10 g L^−1^ |  |  |
|  |  |  | Rep 1 | Rep 2 | Rep 3 | Rep 1 | Rep 2 | Rep 3 | Rep 1 | Rep 2 | Rep 3 | Rep 1 | Rep 2 | Rep 3 | Rep 1 | Rep 2 | Rep 3 |
|  | 10 | Daphniidae | 0 | 0 | 0 | 0 | 0 | 0 | 0 | 0 | 0 | 0 | 0 | 0 | 0 | 0 | 0 |
|  | 14 | Daphniidae | 0 | 0 | 0 | 0 | 0 | 0 | 1 | 0 | 0 | 0 | 0 | 0 | 0 | 0 | 0 |
|  | 17 | Daphniidae | 0 | 0 | 0 | 0 | 0 | 0 | 0 | 0 | 0 | 0 | 0 | 0 | 0 | 0 | 0 |
|  | 21 | Daphniidae | 0 | 0 | 0 | 0 | 0 | 0 | 0 | 0 | 0 | 0 | 0 | 0 | 0 | 0 | 0 |
|  | 24 | Daphniidae | 0 | 0 | 0 | 0 | 0 | 0 | 0 | 0 | 0 | 0 | 0 | 0 | 0 | 0 | 0 |
|  | 28 | Daphniidae | 0 | 0 | 0 | 0 | 0 | 0 | 0 | 0 | 0 | 0 | 0 | 0 | 0 | 0 | 0 |
|  |  |  |  |  |  |  |  |  |  |  |  |  |  |  |  |  |  |
|  | 3 | Ostracoda | 0 | 0 | 0 | 0 | 0 | 0 | 0 | 0 | 0 | 0 | 0 | 0 | 0 | 0 | 0 |
|  | 7 | Ostracoda | 0 | 0 | 0 | 0 | 0 | 0 | 0 | 0 | 0 | 0 | 0 | 0 | 0 | 0 | 0 |
|  | 10 | Ostracoda | 0 | 1 | 0 | 1 | 0 | 1 | 0 | 0 | 1 | 0 | 0 | 0 | 0 | 0 | 0 |
|  | 14 | Ostracoda | 0 | 3 | 0 | 2 | 1 | 1 | 0 | 0 | 1 | 0 | 0 | 0 | 0 | 0 | 0 |
|  | 17 | Ostracoda | 0 | 2 | 2 | 1 | 4 | 3 | 0 | 0 | 1 | 0 | 0 | 0 | 0 | 0 | 0 |
|  | 21 | Ostracoda | 3 | 3 | 2 | 1 | 1 | 3 | 5 | 3 | 2 | 0 | 0 | 0 | 0 | 0 | 0 |
|  | 24 | Ostracoda | 0 | 5 | 1 | 0 | 2 | 3 | 1 | 2 | 4 | 0 | 0 | 1 | 0 | 0 | 0 |
|  | 28 | Ostracoda | 1 | 0 | 4 | 1 | 0 | 7 | 2 | 3 | 3 | 0 | 1 | 0 | 0 | 0 | 0 |
|  |  |  |  |  |  |  |  |  |  |  |  |  |  |  |  |  |  |
| W27B | 3 | Anostraca | 5 | 2 | 3 | 0 | 0 | 0 | 0 | 0 | 0 | 0 | 0 | 0 | 0 | 0 | 0 |
|  | 7 | Anostraca | 4 | 1 | 5 | 1 | 0 | 2 | 0 | 0 | 0 | 0 | 0 | 0 | 0 | 0 | 0 |
|  | 10 | Anostraca | 0 | 15 | 0 | 0 | 0 | 0 | 0 | 0 | 0 | 0 | 0 | 0 | 0 | 0 | 0 |
|  | 14 | Anostraca | 8 | 0 | 1 | 0 | 5 | 1 | 0 | 0 | 0 | 1 | 0 | 0 | 0 | 0 | 0 |
| Code | Day | Taxa | Control |  |  | 0.5 g L^−1^ |  |  | 2.5 g L^−1^ |  |  | 5 g L^−1^ |  |  | 10 g L^−1^ |  |  |
|  |  |  | Rep 1 | Rep 2 | Rep 3 | Rep 1 | Rep 2 | Rep 3 | Rep 1 | Rep 2 | Rep 3 | Rep 1 | Rep 2 | Rep 3 | Rep 1 | Rep 2 | Rep 3 |
|  | 17 | Anostraca | 0 | 1 | 0 | 0 | 0 | 0 | 0 | 0 | 0 | 0 | 0 | 0 | 0 | 0 | 0 |
|  | 21 | Anostraca | 0 | 0 | 0 | 0 | 0 | 0 | 0 | 0 | 0 | 0 | 0 | 0 | 0 | 0 | 0 |
|  | 24 | Anostraca | 0 | 0 | 2 | 0 | 0 | 0 | 0 | 0 | 0 | 0 | 0 | 0 | 0 | 0 | 0 |
|  | 28 | Anostraca | 0 | 0 | 0 | 0 | 0 | 0 | 0 | 0 | 0 | 0 | 0 | 0 | 0 | 0 | 0 |
|  |  |  |  |  |  |  |  |  |  |  |  |  |  |  |  |  |  |
|  | 3 | Copepoda | 2 | 0 | 0 | 0 | 4 | 0 | 0 | 0 | 0 | 0 | 0 | 0 | 0 | 0 | 0 |
|  | 7 | Copepoda | 0 | 0 | 1 | 0 | 1 | 0 | 0 | 0 | 0 | 0 | 0 | 0 | 0 | 0 | 0 |
|  | 10 | Copepoda | 0 | 12 | 0 | 0 | 0 | 0 | 0 | 1 | 0 | 0 | 0 | 0 | 0 | 0 | 0 |
|  | 14 | Copepoda | 4 | 5 | 7 | 0 | 5 | 7 | 0 | 8 | 0 | 0 | 0 | 0 | 0 | 0 | 0 |
|  | 17 | Copepoda | 2 | 0 | 39 | 0 | 0 | 0 | 0 | 0 | 1 | 0 | 0 | 0 | 0 | 0 | 0 |
|  | 21 | Copepoda | 0 | 1 | 0 | 0 | 0 | 0 | 0 | 0 | 0 | 1 | 1 | 1 | 0 | 0 | 0 |
|  | 24 | Copepoda | 0 | 0 | 1 | 0 | 2 | 0 | 0 | 0 | 0 | 0 | 0 | 0 | 0 | 0 | 0 |
|  | 28 | Copepoda | 1 | 3 | 47 | 0 | 7 | 5 | 0 | 1 | 0 | 0 | 0 | 0 | 0 | 0 | 1 |
|  |  |  |  |  |  |  |  |  |  |  |  |  |  |  |  |  |  |
|  | 3 | Spinicaudata | 2 | 0 | 0 | 0 | 0 | 0 | 0 | 0 | 0 | 0 | 0 | 0 | 0 | 0 | 0 |
|  | 7 | Spinicaudata | 1 | 2 | 1 | 0 | 0 | 0 | 0 | 0 | 0 | 0 | 0 | 0 | 0 | 0 | 0 |
|  | 10 | Spinicaudata | 0 | 3 | 0 | 0 | 0 | 0 | 0 | 0 | 0 | 0 | 0 | 0 | 0 | 0 | 0 |
|  | 14 | Spinicaudata | 0 | 0 | 0 | 0 | 0 | 0 | 0 | 0 | 0 | 0 | 0 | 0 | 0 | 0 | 0 |
|  | 17 | Spinicaudata | 0 | 0 | 0 | 0 | 0 | 0 | 0 | 0 | 0 | 0 | 0 | 0 | 0 | 0 | 0 |
|  | 21 | Spinicaudata | 0 | 0 | 0 | 0 | 0 | 0 | 0 | 0 | 0 | 0 | 0 | 0 | 0 | 0 | 0 |
| Code | Day | Taxa | Control |  |  | 0.5 g L^−1^ |  |  | 2.5 g L^−1^ |  |  | 5 g L^−1^ |  |  | 10 g L^−1^ |  |  |
|  |  |  | Rep 1 | Rep 2 | Rep 3 | Rep 1 | Rep 2 | Rep 3 | Rep 1 | Rep 2 | Rep 3 | Rep 1 | Rep 2 | Rep 3 | Rep 1 | Rep 2 | Rep 3 |
|  | 24 | Spinicaudata | 0 | 0 | 0 | 0 | 0 | 0 | 0 | 0 | 0 | 0 | 0 | 0 | 0 | 0 | 0 |
|  | 28 | Spinicaudata | 0 | 1 | 0 | 0 | 0 | 0 | 0 | 0 | 0 | 0 | 0 | 0 | 0 | 0 | 0 |
|  |  |  |  |  |  |  |  |  |  |  |  |  |  |  |  |  |  |
|  | 3 | Notostraca | 7 | 9 | 0 | 4 | 9 | 0 | 0 | 0 | 0 | 0 | 0 | 0 | 0 | 0 | 0 |
|  | 7 | Notostraca | 2 | 3 | 0 | 0 | 0 | 1 | 0 | 0 | 0 | 0 | 0 | 0 | 0 | 0 | 0 |
|  | 10 | Notostraca | 0 | 4 | 1 | 1 | 1 | 0 | 0 | 1 | 3 | 0 | 0 | 0 | 0 | 0 | 0 |
|  | 14 | Notostraca | 1 | 0 | 0 | 2 | 0 | 0 | 0 | 0 | 2 | 0 | 0 | 0 | 0 | 0 | 0 |
|  | 17 | Notostraca | 3 | 1 | 0 | 0 | 0 | 0 | 0 | 0 | 0 | 1 | 0 | 0 | 0 | 0 | 0 |
|  | 21 | Notostraca | 0 | 0 | 0 | 0 | 0 | 1 | 0 | 0 | 0 | 0 | 0 | 0 | 0 | 0 | 0 |
|  | 24 | Notostraca | 0 | 0 | 0 | 0 | 0 | 0 | 0 | 0 | 0 | 0 | 0 | 0 | 0 | 0 | 0 |
|  | 28 | Notostraca | 0 | 1 | 0 | 0 | 0 | 0 | 0 | 0 | 0 | 0 | 0 | 0 | 0 | 0 | 0 |
|  |  |  |  |  |  |  |  |  |  |  |  |  |  |  |  |  |  |
|  | 3 | Daphniidae | 0 | 0 | 0 | 0 | 0 | 0 | 0 | 0 | 0 | 0 | 0 | 0 | 0 | 0 | 0 |
|  | 7 | Daphniidae | 0 | 0 | 0 | 0 | 0 | 0 | 0 | 0 | 0 | 0 | 0 | 0 | 0 | 0 | 0 |
|  | 10 | Daphniidae | 0 | 0 | 0 | 0 | 0 | 0 | 0 | 0 | 0 | 0 | 0 | 0 | 0 | 0 | 0 |
|  | 14 | Daphniidae | 2 | 0 | 0 | 1 | 0 | 2 | 0 | 0 | 0 | 0 | 0 | 0 | 0 | 0 | 0 |
|  | 17 | Daphniidae | 0 | 0 | 0 | 0 | 0 | 0 | 0 | 0 | 0 | 1 | 0 | 0 | 0 | 0 | 0 |
|  | 21 | Daphniidae | 0 | 0 | 0 | 0 | 0 | 0 | 0 | 0 | 0 | 0 | 0 | 0 | 0 | 0 | 0 |
|  | 24 | Daphniidae | 0 | 0 | 0 | 0 | 0 | 0 | 0 | 0 | 0 | 0 | 0 | 0 | 0 | 0 | 0 |
|  | 28 | Daphniidae | 0 | 0 | 0 | 0 | 0 | 0 | 0 | 0 | 0 | 0 | 0 | 0 | 0 | 0 | 0 |
| Code | Day | Taxa | Control |  |  | 0.5 g L^−1^ |  |  | 2.5 g L^−1^ |  |  | 5 g L^−1^ |  |  | 10 g L^−1^ |  |  |
|  |  |  | Rep 1 | Rep 2 | Rep 3 | Rep 1 | Rep 2 | Rep 3 | Rep 1 | Rep 2 | Rep 3 | Rep 1 | Rep 2 | Rep 3 | Rep 1 | Rep 2 | Rep 3 |
|  | 3 | Ostracoda | 0 | 0 | 0 | 0 | 0 | 0 | 0 | 0 | 0 | 0 | 0 | 0 | 0 | 0 | 0 |
|  | 7 | Ostracoda | 0 | 0 | 0 | 0 | 0 | 0 | 0 | 0 | 0 | 0 | 0 | 0 | 0 | 0 | 0 |
|  | 10 | Ostracoda | 0 | 8 | 0 | 2 | 0 | 0 | 0 | 0 | 0 | 0 | 0 | 0 | 0 | 0 | 0 |
|  | 14 | Ostracoda | 16 | 26 | 21 | 0 | 7 | 11 | 0 | 0 | 0 | 0 | 0 | 0 | 0 | 0 | 0 |
|  | 17 | Ostracoda | 14 | 11 | 5 | 1 | 9 | 5 | 6 | 15 | 2 | 0 | 0 | 0 | 0 | 0 | 0 |
|  | 21 | Ostracoda | 8 | 3 | 6 | 14 | 10 | 7 | 0 | 10 | 0 | 0 | 0 | 0 | 0 | 0 | 0 |
|  | 24 | Ostracoda | 3 | 5 | 0 | 6 | 3 | 2 | 0 | 3 | 2 | 0 | 0 | 2 | 0 | 0 | 1 |
|  | 28 | Ostracoda | 17 | 27 | 17 | 16 | 21 | 15 | 2 | 21 | 2 | 0 | 0 | 2 | 0 | 0 | 0 |
|  |  |  |  |  |  |  |  |  |  |  |  |  |  |  |  |  |  |
| W110 | 3 | Anostraca | 0 | 0 | 0 | 0 | 0 | 0 | 0 | 0 | 0 | 0 | 0 | 0 | 0 | 0 | 0 |
|  | 7 | Anostraca | 0 | 0 | 0 | 0 | 0 | 0 | 0 | 0 | 0 | 0 | 0 | 0 | 0 | 0 | 0 |
|  | 10 | Anostraca | 2 | 0 | 3 | 0 | 9 | 0 | 0 | 1 | 0 | 0 | 0 | 0 | 0 | 0 | 0 |
|  | 14 | Anostraca | 0 | 0 | 0 | 0 | 0 | 0 | 0 | 0 | 1 | 0 | 0 | 1 | 0 | 0 | 0 |
|  | 17 | Anostraca | 0 | 0 | 0 | 0 | 0 | 0 | 0 | 0 | 0 | 0 | 0 | 0 | 0 | 0 | 0 |
|  | 21 | Anostraca | 0 | 0 | 0 | 0 | 0 | 0 | 0 | 0 | 0 | 0 | 0 | 0 | 0 | 0 | 0 |
|  | 24 | Anostraca | 1 | 0 | 0 | 0 | 0 | 0 | 0 | 0 | 0 | 0 | 0 | 0 | 0 | 0 | 0 |
|  | 28 | Anostraca | 0 | 0 | 0 | 0 | 0 | 0 | 0 | 0 | 0 | 0 | 0 | 0 | 0 | 0 | 0 |
|  |  |  |  |  |  |  |  |  |  |  |  |  |  |  |  |  |  |
|  | 3 | Copepoda | 0 | 0 | 0 | 0 | 0 | 0 | 0 | 0 | 0 | 0 | 0 | 0 | 0 | 0 | 0 |
|  | 7 | Copepoda | 0 | 0 | 0 | 0 | 0 | 0 | 0 | 0 | 0 | 0 | 0 | 0 | 0 | 0 | 0 |
| Code | Day | Taxa | Control |  |  | 0.5 g L^−1^ |  |  | 2.5 g L^−1^ |  |  | 5 g L^−1^ |  |  | 10 g L^−1^ |  |  |
|  |  |  | Rep 1 | Rep 2 | Rep 3 | Rep 1 | Rep 2 | Rep 3 | Rep 1 | Rep 2 | Rep 3 | Rep 1 | Rep 2 | Rep 3 | Rep 1 | Rep 2 | Rep 3 |
|  | 10 | Copepoda | 3 | 0 | 3 | 0 | 1 | 0 | 0 | 2 | 0 | 0 | 0 | 0 | 0 | 0 | 0 |
|  | 14 | Copepoda | 0 | 3 | 9 | 2 | 7 | 0 | 0 | 3 | 1 | 0 | 2 | 0 | 0 | 0 | 0 |
|  | 17 | Copepoda | 4 | 8 | 7 | 1 | 1 | 1 | 0 | 0 | 0 | 0 | 2 | 1 | 1 | 0 | 0 |
|  | 21 | Copepoda | 0 | 12 | 17 | 2 | 3 | 1 | 0 | 1 | 2 | 3 | 0 | 5 | 0 | 0 | 0 |
|  | 24 | Copepoda | 12 | 0 | 27 | 0 | 0 | 0 | 1 | 1 | 0 | 0 | 1 | 1 | 1 | 0 | 0 |
|  | 28 | Copepoda | 0 | 71 | 6 | 5 | 0 | 0 | 0 | 0 | 0 | 1 | 0 | 0 | 0 | 0 | 0 |
|  |  |  |  |  |  |  |  |  |  |  |  |  |  |  |  |  |  |
|  | 3 | Spinicaudata | 0 | 0 | 0 | 0 | 0 | 0 | 0 | 0 | 0 | 0 | 0 | 0 | 0 | 0 | 0 |
|  | 7 | Spinicaudata | 0 | 0 | 0 | 0 | 0 | 0 | 0 | 0 | 0 | 0 | 0 | 0 | 0 | 0 | 0 |
|  | 10 | Spinicaudata | 0 | 0 | 2 | 0 | 0 | 0 | 0 | 0 | 0 | 0 | 0 | 0 | 0 | 0 | 0 |
|  | 14 | Spinicaudata | 0 | 0 | 0 | 0 | 0 | 0 | 0 | 0 | 0 | 0 | 0 | 0 | 0 | 0 | 0 |
|  | 17 | Spinicaudata | 0 | 0 | 0 | 0 | 0 | 0 | 0 | 0 | 0 | 0 | 0 | 0 | 0 | 0 | 0 |
|  | 21 | Spinicaudata | 0 | 0 | 0 | 0 | 0 | 0 | 0 | 0 | 0 | 0 | 0 | 0 | 0 | 0 | 0 |
|  | 24 | Spinicaudata | 0 | 0 | 0 | 0 | 0 | 0 | 0 | 0 | 0 | 0 | 0 | 0 | 0 | 0 | 0 |
|  | 28 | Spinicaudata | 0 | 0 | 0 | 0 | 0 | 0 | 0 | 0 | 0 | 0 | 0 | 0 | 0 | 0 | 0 |
|  |  |  |  |  |  |  |  |  |  |  |  |  |  |  |  |  |  |
|  | 3 | Notostraca | 0 | 0 | 0 | 0 | 0 | 0 | 0 | 0 | 0 | 0 | 0 | 0 | 0 | 0 | 0 |
|  | 7 | Notostraca | 1 | 0 | 0 | 0 | 0 | 0 | 0 | 0 | 0 | 0 | 0 | 0 | 0 | 0 | 0 |
|  | 10 | Notostraca | 4 | 0 | 0 | 0 | 0 | 0 | 0 | 0 | 0 | 0 | 0 | 0 | 0 | 0 | 0 |
|  | 14 | Notostraca | 0 | 0 | 0 | 0 | 0 | 0 | 0 | 0 | 0 | 0 | 6 | 0 | 0 | 0 | 0 |
| Code | Day | Taxa | Control |  |  | 0.5 g L^−1^ |  |  | 2.5 g L^−1^ |  |  | 5 g L^−1^ |  |  | 10 g L^−1^ |  |  |
|  |  |  | Rep 1 | Rep 2 | Rep 3 | Rep 1 | Rep 2 | Rep 3 | Rep 1 | Rep 2 | Rep 3 | Rep 1 | Rep 2 | Rep 3 | Rep 1 | Rep 2 | Rep 3 |
|  | 17 | Notostraca | 0 | 0 | 0 | 0 | 0 | 0 | 0 | 0 | 0 | 0 | 0 | 0 | 0 | 0 | 0 |
|  | 21 | Notostraca | 0 | 0 | 0 | 0 | 0 | 0 | 0 | 0 | 0 | 0 | 0 | 0 | 0 | 0 | 0 |
|  | 24 | Notostraca | 0 | 0 | 0 | 0 | 0 | 0 | 0 | 0 | 0 | 0 | 0 | 0 | 0 | 0 | 0 |
|  | 28 | Notostraca | 0 | 0 | 0 | 0 | 0 | 0 | 0 | 0 | 0 | 0 | 0 | 0 | 0 | 0 | 0 |
|  |  |  |  |  |  |  |  |  |  |  |  |  |  |  |  |  |  |
|  | 3 | Daphniidae | 0 | 0 | 0 | 0 | 0 | 0 | 0 | 0 | 0 | 0 | 0 | 0 | 0 | 0 | 0 |
|  | 7 | Daphniidae | 0 | 0 | 0 | 0 | 0 | 0 | 0 | 0 | 0 | 0 | 0 | 0 | 0 | 0 | 0 |
|  | 10 | Daphniidae | 3 | 0 | 5 | 0 | 5 | 0 | 0 | 3 | 0 | 0 | 0 | 0 | 0 | 0 | 0 |
|  | 14 | Daphniidae | 2 | 8 | 9 | 3 | 2 | 0 | 1 | 0 | 0 | 0 | 0 | 0 | 0 | 0 | 0 |
|  | 17 | Daphniidae | 3 | 2 | 1 | 1 | 8 | 13 | 1 | 3 | 0 | 0 | 2 | 0 | 0 | 0 | 0 |
|  | 21 | Daphniidae | 0 | 0 | 0 | 1 | 2 | 17 | 11 | 1 | 0 | 0 | 4 | 1 | 0 | 0 | 0 |
|  | 24 | Daphniidae | 0 | 0 | 0 | 3 | 2 | 39 | 9 | 1 | 1 | 0 | 0 | 0 | 0 | 0 | 0 |
|  | 28 | Daphniidae | 0 | 0 | 0 | 0 | 91 | 14 | 16 | 3 | 0 | 0 | 1 | 0 | 0 | 0 | 0 |
|  |  |  |  |  |  |  |  |  |  |  |  |  |  |  |  |  |  |
|  | 3 | Ostracoda | 0 | 0 | 0 | 0 | 0 | 0 | 0 | 0 | 0 | 0 | 0 | 0 | 0 | 0 | 0 |
|  | 7 | Ostracoda | 0 | 0 | 0 | 0 | 0 | 0 | 0 | 0 | 0 | 0 | 0 | 0 | 0 | 0 | 0 |
|  | 10 | Ostracoda | 0 | 0 | 0 | 0 | 0 | 0 | 0 | 0 | 0 | 0 | 0 | 0 | 0 | 0 | 0 |
|  | 14 | Ostracoda | 0 | 0 | 01 | 0 | 2 | 0 | 0 | 1 | 0 | 0 | 0 | 0 | 0 | 0 | 0 |
|  | 17 | Ostracoda | 1 | 6 | 4 | 0 | 1 | 1 | 0 | 0 | 0 | 2 | 0 | 0 | 1 | 1 | 0 |
|  | 21 | Ostracoda | 3 | 2 | 7 | 3 | 7 | 2 | 0 | 3 | 1 | 1 | 0 | 1 | 0 | 0 | 0 |
| Code | Day | Taxa | Control |  |  | 0.5 g L^−1^ |  |  | 2.5 g L^−1^ |  |  | 5 g L^−1^ |  |  | 10 g L^−1^ |  |  |
|  |  |  | Rep 1 | Rep 2 | Rep 3 | Rep 1 | Rep 2 | Rep 3 | Rep 1 | Rep 2 | Rep 3 | Rep 1 | Rep 2 | Rep 3 | Rep 1 | Rep 2 | Rep 3 |
|  | 24 | Ostracoda | 2 | 2 | 13 | 0 | 8 | 7 | 4 | 8 | 6 | 0 | 0 | 2 | 0 | 0 | 0 |
|  | 28 | Ostracoda | 1 | 16 | 77 | 33 | 59 | 57 | 24 | 16 | 6 | 1 | 4 | 15 | 1 | 0 | 5 |

**Table S2**. **Hatchling abundance data (presented for each taxa) during the 28-days inundation period of sediments from four depression wetlands from the semi-arid Eastern Cape Karoo region.** Rep = replicate.
